# Diagnostic Efficacy of Ultra-Short Term HRV Analysis in Obstructive Sleep Apnea

**DOI:** 10.3390/jpm12091494

**Published:** 2022-09-13

**Authors:** Seung-Su Ha, Dong-Kyu Kim

**Affiliations:** 1 Department of Otorhinolaryngology-Head and Neck Surgery, Chuncheon Sacred Heart Hospital, Hallym University College of Medicine, Chuncheon 24253, Korea; 2 Institute of New Frontier Research, Division of Big Data and Artificial Intelligence, Chuncheon Sacred Heart Hospital, Hallym University College of Medicine, Chuncheon 24253, Korea

**Keywords:** heart rate variability, autonomic nervous system, sleep, obstructive sleep apnea

## Abstract

Heart rate variability (HRV) is the standard method for assessing autonomic nervous system (ANS) activity and is considered a surrogate marker for sympathetic overactivity in obstructive sleep apnea (OSA). Although HRV features are usually obtained from the short-term segment method, it is impossible to evaluate rapid dynamic changes in ANS activity. Herein, we propose the ultra-short-term analysis to detect the balance of ANS activity in patients with OSA. In 1021 OSA patients, 10 min HRV target datasets were extracted from polysomnographic data and analyzed by shifting the 2 min (ultra-short-term) and 5 min (short-term) segments. We detected frequency-domain parameters, including total power (Ln TP), very low frequency (Ln VLF), low frequency (Ln LF), and high frequency (Ln HF). We found that overall HRV feature alterations indicated sympathetic overactivity dependent on OSA severity, and that this was more pronounced in the ultra-short-term methodology. The apnea-hypopnea index, oxygen desaturation index, and Epworth sleepiness scale correlated with increased sympathetic activity and decreased parasympathetic activity, regardless of the methodology. The Bland-Altman plot analyses also showed a higher agreement of HRV features between the two methodologies. This study suggests that ultra-short-term HRV analysis may be a useful method for detecting alterations in ANS function in OSA patients.

## 1. Introduction

Obstructive sleep apnea (OSA) is the most common sleep-related breathing disorder and is characterized by total or partial upper airway collapse. Patients with OSA usually experience frequent hypoxic events and sleep fragmentation, resulting in repeated exposure to hypoxemia and hypercapnia. Additionally, this phenomenon could induce sympathetic overactivity in patients [1,2,3]. These alterations in autonomic nervous system (ANS) activity also contribute to the development of cardiovascular diseases in patients with OSA [4,5,6]. Heart rate variability (HRV) is a noninvasive method for the assessment of changes in ANS activity and represents the balance between the parasympathetic nervous system (PNS) and sympathetic nervous system (SNS) [7]. Most studies regarding HRV have shown a higher low-frequency power (LF) and a higher ratio of LF to high-frequency power (HF) in patients with OSA than in healthy subjects [8,9,10]. These findings support the idea that the balance shift in ANS activity moves toward sympathetic overactivity in these patients. In fact, evidence suggests that HRV can assess the shift in ANS activity and could be a useful marker of OSA severity [11,12,13,14].

To date, linear and non-linear methods of HRV analysis have been used. There are several algorithms of the non-linear method, such as Hurst exponent and Higuchi fractal dimension. However, HRV analysis is usually performed as a function of the time or temporal frequencies from linear analysis methods: peaks below 0.04 Hz (very low frequency, VLF), between 0.04 and 0.15 Hz (low frequency, LF), and between 0.15 and 0.4 (high frequency, HF). Previously, the majority of these studies used a short-term window, which investigates the degree of fluctuations in the time and frequency domains, to evaluate the HRV analysis. Short-term segment HRV is typically calculated over 5 min, and the magnitude of the frequency components obtained by spectral analysis is useful for assessing ANS activity [15]. However, HRV analysis based on short-term segments has inevitable limitations in clinical applications because its spectral estimates cannot reflect rapid ANS fluctuations. For this reason, several recent studies have suggested that ultra-short-term segments (shorter than 5 min) may be effective alternatives to short-term HRV analysis for mental stress detection [16,17,18]. Herein, we investigated the frequency spectral analysis of HRV features using the ultra-short-term methodology and evaluated whether the methodology could be more effective as a diagnostic marker for the alteration of ANS activity in patients with OSA. To the best of our knowledge, no studies have investigated ultra-short HRV as a valid surrogate in patients with OSA.

## 2. Materials and Methods

We consecutively enrolled patients diagnosed with OSA at the Sleep Center of Hallym Medical University Chuncheon Sacred Hospital and the informed consent was obtained from all subjects involved in the study. We excluded patients who had previously been diagnosed with a psychological disorder, a history of any medical condition that could influence sympathovagal activity, and serious comorbidities (e.g., cancer, severe depression or insomnia, severe cardiac or respiratory failure, and severe renal or hepatic insufficiency). We performed overnight full polysomnography (PSG) and obtained the Epworth Sleepiness Scale (ESS) scores for all enrolled patients to assess daytime sleepiness. In this study, sleep stage and respiratory events were scored according to the American Academy of Sleep Medicine [19]. The following sleep parameters were assessed: total sleep time (TST), sleep efficiency (TST/time in bed × 100), sleep latency, oxygen desaturation index (ODI), and apnea-hypopnea index (AHI). ODI was defined as the number of episodes of oxygen desaturation per hour of sleep, with oxygen desaturation defined as a decrease in blood oxygen saturation (SpO_2_) to lower than 3% below baseline. The AHI was defined as the sum of the number of apneas and hypopneas per hour of sleep. In this study, we defined apnea as when the peak signal excursions, measured using an oronasal thermal sensor, dropped by ≥90% of baseline before the event, lasting for more than 10 s. Conversely, hypopnea was scored when the peak signal excursions using nasal pressure dropped by ≥30% of baseline before the event for at least 10 s, followed by a ≥ 3% decrease in oxygen desaturation or accompanied by arousal. Based on AHI, we classified the severity of OSA as follows: normal (AHI < 5), mild OSA (5 ≤ AHI < 15), moderate OSA (15 ≤ AHI < 30), and severe OSA (AHI ≥ 30).

HRV is characterized by the variability in beat-to-beat intervals of the heart and is typically measured using RR intervals extracted from an ECG signal. Thus, in this study, we extracted the electrocardiography data from PSG recordings visually inspected for accuracy and quality. Specifically, we eliminated ectopic beats and artifacts and selected only normal-to-normal beats for the HRV analysis. Generally, time-domain HRV parameters showed variability in beat-to-beat intervals, which influenced both sympathetic and parasympathetic activities [20]. Thus, these parameters are not typically used to discriminate the balance of ANS activity (sympathetic and parasympathetic functions). For this reason, we obtained the frequency-domain HRV parameters. We used four spectral power analyses by shifting predetermined segments (ultra-short-term, 2 min and short-term, 5 min) forward by 2 s during the entire 10 min HRV target dataset, which were collected from each patient: total power (Ln TP), very low frequency (Ln VLF), low frequency (Ln LF), and high frequency (Ln HF) in frequency-domain measures.

Numerical variables are expressed as mean ± standard deviation, and Student’s *t*-test was used to compare HRV parameters after the normality test. ANOVA with post hoc analysis was performed to compare the specific values according to the OSA severity group. Pearson’s coefficient test was used to identify the relationships between HRV and sleep parameters according to OSA severity. To compare the agreement of the HRV parameters between the ultra-short-term and short-term segments, we used Bland-Altman plots. All statistical analyses were conducted using R version 3.5.0 (R Foundation for Statistical Computing, Vienna, Austria). Statistical significance was set at *p* < 0.05.

## 3. Results

In total, 1021 patients with OSA were enrolled in this study. The patients were divided into three groups: mild OSA (*n* = 238), moderate OSA (*n* = 331), and severe OSA (*n* = 452). The demographics and PSG parameters are presented in Table 1. Age and ESS were significantly higher in patients with severe OSA than in the other groups (*p* < 0.001), whereas there were no significant group differences in TST, sleep latency, and sleep efficiency.

A 10 min HRV target segment was divided into consecutive individual ultra-short-term and short-term segments every 2 s, and all frequency-domain HRV parameters were averaged according to OSA severity (Table 2, Table 3 and Table 4). In mild and moderate OSA, Ln TP and Ln HF were significantly higher in the ultra-short term than in the short term (Table 2 and Table 3), whereas the values of Ln TP, Ln VLF, and Ln LF based on the ultra-short term were significantly higher in those based on the short term (Table 4). However, there was no significant difference in the value of Ln LF/Ln HF between the ultra-short-term and short-term segments, regardless of OSA severity. We also confirmed that patients with OSA had overall alterations in HRV measures, indicating sympathetic overactivity, and this tendency was more pronounced in those with severe OSA. Additionally, these findings were more clearly detected in the ultra-short-term analysis than in the short-term analysis.

**Figure 1:**
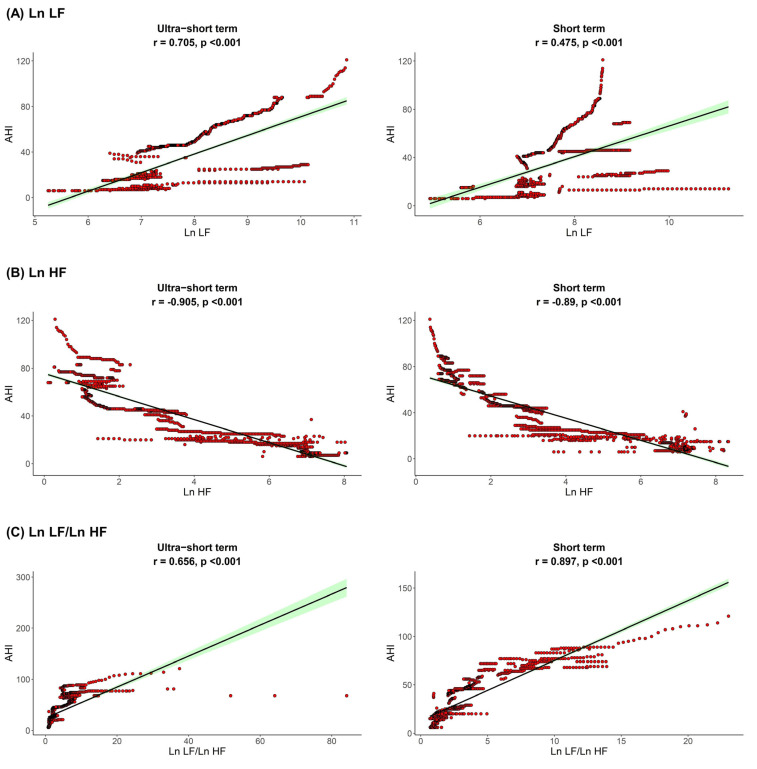
Correlation between AHI (**A**) Ln LF, (**B**) Ln HF, (**C**) Ln LF/Ln HF. Apnea-hypopnea index, AHI; total power, Ln TP; very low frequency, Ln VLF; low frequency, Ln LF; high frequency, Ln HF.

**Figure 2:**
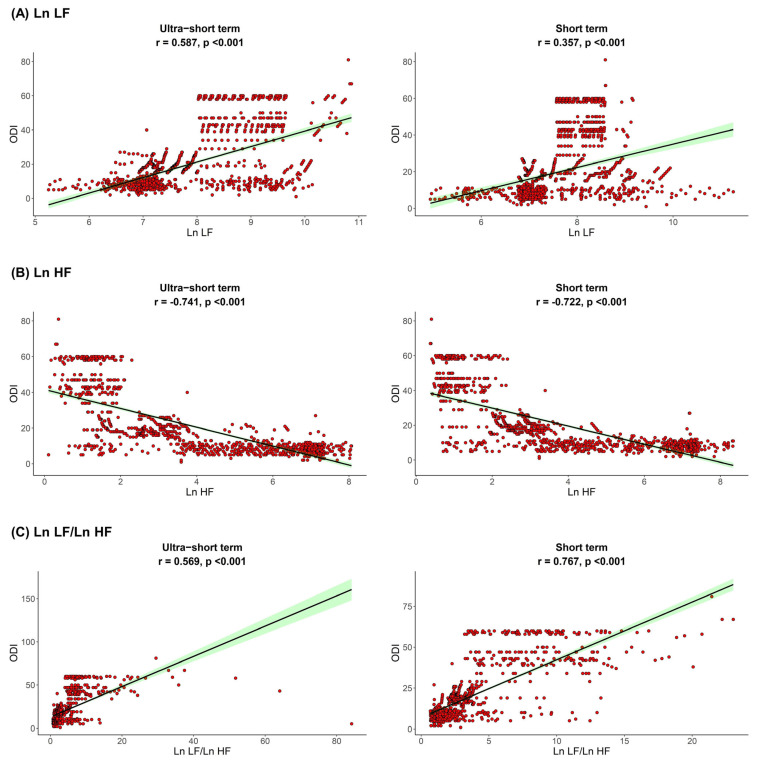
Correlation between ODI and (**A**) Ln LF, (**B**) Ln HF, (**C**) Ln LF/Ln HF. Oxygen desaturation index, ODI; total power, Ln TP; very low frequency, Ln VLF; low frequency, Ln LF; high frequency, Ln HF.

**Figure 3:**
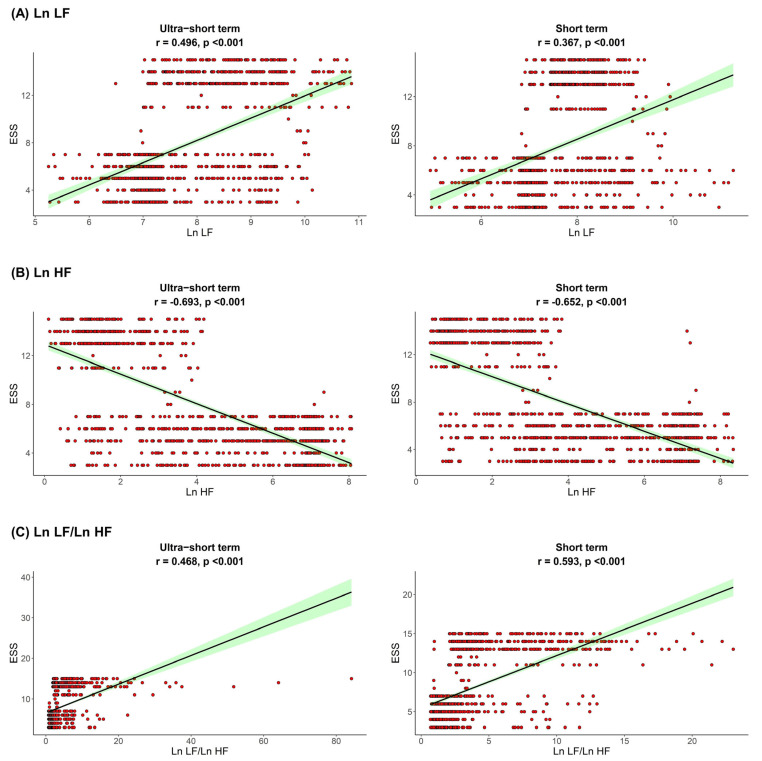
Correlation between ESS and (**A**) Ln LF, (**B**) Ln HF, (**C**) Ln LF/Ln H. Epworth sleepiness scale ESS, total power; Ln TP, very low frequency; Ln VLF, low frequency; Ln LF, high frequency; Ln HF, low frequency.

The correlation between sleep parameters and HRV parameters is shown in Figure 1, Figure 2 and Figure 3. We found that, in both HRV analyses (ultra-short or short-term segments), AHI was positively correlated with Ln LF and Ln LF/Ln HF, whereas there was a negative correlation between AHI and Ln HF. This trend was also detected in the ODI and ESS, although the Pearson’s correlation coefficients slightly decreased from AHI to the ODI and ESS. This means that AHI, ODI, and ESS were well correlated with increased sympathetic activity and decreased parasympathetic activity, a tendency that was more prominent in the ultra-short-term analysis.

**Figure 4:**
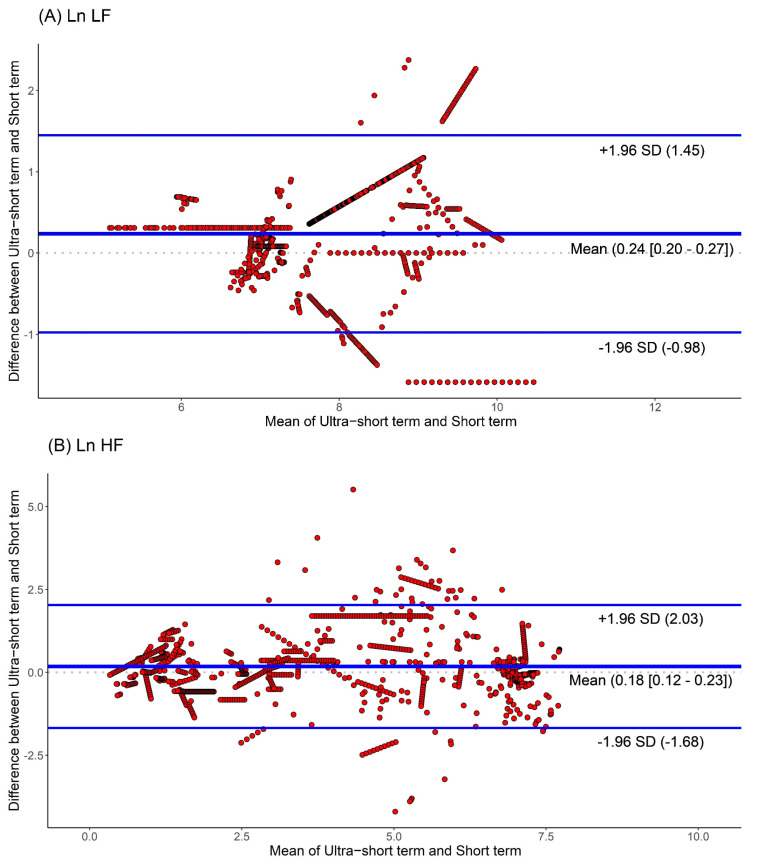

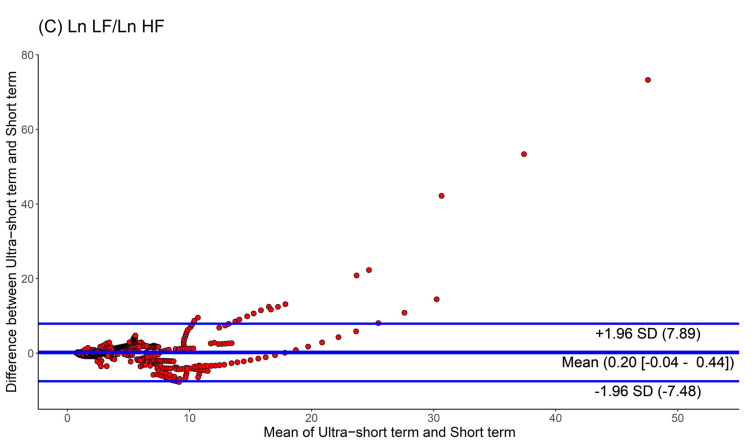
Agreement rate of heart rate variability parameters between ultra-short- and short-term methodology using Bland-Altman plots: (**A**) Ln LF, (**B**) Ln HF, (**C**) Ln LF/Ln HF (standard deviation, SD; low frequency, Ln LF; high frequency, Ln HF).

Moreover, we used Bland-Altman plots to analyze the consistency of the HRV parameters. The Bland-Altman plots for HRV analyses based on ultra-short and short-term segments are shown in Figure 4. We found that the major HRV parameters, such as Ln LF, Ln HF, and Ln LF/Ln HF, were mostly located within the upper and lower 2.0 SD lines. This means that the agreement of HRV parameters between the ultra-short and short-term segments was substantial.

## 4. Discussion

HRV is widely used as a marker related to the ANS and is classically divided into two categories based on the length of the data recording. One is short-term HRV, typically calculated over 5 min, and the magnitude of its frequency components obtained by spectral analysis is used for the assessment of ANS function [21,22,23,24]. Another is long-term HRV computed over a nominal 24 h and its time-frequency domain is used mainly for mortality risk prediction [25,26,27,28]. However, the demand for rapid detection of ANS alterations has increased in several medical areas with the development of wearable devices [29,30]. Thus, ultra-short-term HRV analysis has been actively applied in various medical fields [16,17,31,32,33]. In the present study, to overcome the shortcomings of short-term methodology in HRV analysis, we investigated the effectiveness of ultra-short-term methodology in the detection of ANS alterations in patients with OSA. We found that, in both ultra-short- and short-term methodologies, all HRV parameters showed a similar tendency, which indicated sympathetic overactivity along with increased OSA severity. Additionally, we detected a significant correlation between HRV parameters and AHI, ODI, and ESS scores. The HRV feature also showed a higher agreement between the ultra-short- and short-term methodologies.

The frequency analysis of HRV typically uses three parameters: Ln VLF, Ln LF, and Ln HF. Additionally, Ln TP can be measured by the sum of the total spectral power, and it is thought to represent the global autonomic function. The Ln LF/HF ratio, as a major marker for sympathovagal balance, can also be calculated during HRV analysis. Among HRV studies of OSA, there is widespread evidence demonstrating that the alteration of ANS balance toward sympathetic predominance is well reflected in the Ln LF/HF ratio, Ln LF [8,10,34,35,36]. In addition, a shift in ANS function towards parasympathetic predominance was observed with increases in Ln HF [34,35,36]. Thus, the shift toward sympathetic predominance demonstrated by HRV analysis of patients with OSA could suggest a higher cardiovascular risk during waking status.

It is well known that HRV features obtained from short-term segments show smoother spectral profiles than those obtained from ultra-short-term segments. Thus, short-term methodology may be easy to evaluate in an entirely autonomic state during sleep. However, short-term HRV analysis has inherent limitations in real-world settings. First, short-term HRV-based studies are highly susceptible to noise. Second their failure to detect dynamic ANS activity, especially the rapid changes in Ln LF and Ln HF. However, ultra-short-term HRV analysis could assess the immediate changes in RR intervals on electrocardiograms before and after the period of respiratory events. For these reasons, to investigate sophisticated sleep studies, short-term HRV analysis is not recommended. Although ultra-short-term HRV analyses have not yet been adopted as a standard tool in OSA, a recent study showed that higher ultra-short-term HRV was strongly related to longer respiratory event durations [32]. In this study, we detected substantial agreement in HRV parameters between ultra-short and short-term methodologies. Interestingly, this alteration in HRV value was more prominent with the ultra-short-term method, regardless of OSA severity. Consistent with our findings, ultra-short-term HRV parameters were generally higher with longer respiratory events, regardless of the respiratory event type [37]. Therefore, our findings may be used as a diagnostic marker for the alteration of ANS activity in patients with OSA.

However, our study had several limitations. First, only time-domain HRV parameters were assessed for ANS function, because frequency-domain HRV parameters require a longer RR interval segment for the analysis. Second, this study did not include data on HRV parameters in control subjects. However, to include the control group, we should match the age, sex, BMI, and comorbidities; thus, it is very difficult in the real-world study. Third, in this study, we obtained HRV features from only the linear method, not using the non-linear method. Thus, our further study should include the comparison of HRV features between the linear and the non-linear methodologies. Fourth, several comorbidities could influence the inflammatory status of OSA patients and these conditions could be confounding factors in the HRV analysis [38,39]. Finally, our data need to be used to further evaluate the effect of OSA treatment on HRV features.

## 5. Conclusions

In conclusion, ultra-short-term HRV features are valid surrogates of short-term HRV features for investigating ANS function in OSA patients. Therefore, this study provides valuable insights into the balance of ANS activity in OSA patients.

## Figures and Tables

**Table 1:** Baseline characteristics and polysomnographic parameters.

	Mild	Moderate	Severe
Number	238 (23.3%)	331 (32.4%)	452 (44.3%)
Age	53.87 ± 7.55	56.14 ± 7.91	57.06 ± 8.53
Sex			
Male	206 (86.6%)	237 (71.6%)	403 (89.2%)
Female	32 (13.4%)	94 (28.4%)	49 (10.8%)
Body mass index (kg/m^2^)	24.2 ± 3.8	26.4 ± 4.8	27.2 ± 3.1
Hypertension	168	216	285
ESS	5.06 ± 1.34	5.56 ± 2.24	11.11 ± 4.04
Total sleep Time (min)	363.93 ± 56.94	367.12 ± 56.43	375.67 ± 55.23
Sleep latency (min)	44.70 ± 9.64	45.94 ± 8.19	45.76 ± 7.72
Sleep efficiency (%)	79.74 ± 6.63	79.59 ± 6.42	79.30 ± 6.53
AHI	9.34 ± 2.37	20.50 ± 3.86	60.69 ± 17.38
ODI	7.87 ± 2.23	9.43 ± 3.96	33.62 ± 17.21

Epworth sleepiness scale, ESS; apnea-hypopnea index, (AHI); oxygen desaturation index, (ODI).

**Table 2:** Heart rate variability parameters between ultra-short and short term in mild obstructive sleep apnea.

	Ultra-Short Term	Short Term	*p* Value
Ln TP	7.47 ± 0.34	7.29 ± 0.47	<0.001
Ln VLF	6.97 ± 0.57	6.93 ± 0.61	0.504
Ln LF	7.24 ± 0.91	7.20 ± 1.21	0.733
Ln HF	7.10 ± 0.29	6.97 ± 0.61	0.002
Ln LF/Ln HF	1.02 ± 0.15	1.04 ± 0.20	0.221

Total power, Ln TP; very low frequency, Ln VLF; low frequency, Ln LF; high frequency, Ln HF.

**Table 3:** Heart rate variability parameters between ultra-short and short term in moderate obstructive sleep apnea.

	Ultra-Short Term	Short Term	*p* Value
Ln TP	7.43 ± 0.35	7.30 ± 0.46	<0.001
Ln VLF	7.19 ± 0.58	7.13 ± 0.53	0.152
Ln LF	7.56 ± 1.08	7.41 ± 1.00	0.071
Ln HF	5.18 ± 1.24	4.71 ± 1.45	<0.001
Ln LF/Ln HF	1.60 ± 0.64	1.78 ± 0.75	0.001

Total power, Ln TP; very low frequency, Ln VLF; low frequency, Ln LF; high frequency, Ln HF.

**Table 4:** Heart rate variability parameters between ultra-short and short term in severe obstructive sleep apnea.

	Ultra-Short Term	Short Term	*p* Value
Ln TP	7.45 ± 0.34	7.22 ± 0.50	<0.001
Ln VLF	7.19 ± 0.52	7.02 ± 0.58	<0.001
Ln LF	8.36 ± 0.97	7.95 ± 0.57	<0.001
Ln HF	1.80 ± 0.92	1.82 ± 1.06	0.849
Ln LF/Ln HF	6.93 ± 7.17	6.33 ± 4.14	0.125

Total power, Ln TP; very low frequency, Ln VLF; low frequency, Ln LF; high frequency, Ln HF.

## Data Availability

The authors confirm that data supporting the findings of this study are available within the article.
